# Laryngeal Tuberculosis Mimicking Laryngeal Carcinoma on ^18^F-FDG PET/CT Imaging

**DOI:** 10.4274/mirt.44366

**Published:** 2018-06-07

**Authors:** Arzu Cengiz, Sibel Göksel, Yeşim Başal, Şule Taş Gülen, Füruzan Döğer, Yakup Yürekli

**Affiliations:** 1 Adnan Menderes University Faculty of Medicine, Department of Nuclear Medicine, Aydın, Turkey; 2 Adnan Menderes University Faculty of Medicine, Department of Otorhinolaryngology, Aydın, Turkey; 3 Adnan Menderes University Faculty of Medicine, Department of Chest Diseases, Aydın, Turkey; 4 Adnan Menderes University Faculty of Medicine, Department of Pathology, Aydın, Turkey

**Keywords:** Tuberculosis, laryngeal cancer, positron emission tomography/computed tomography

## Abstract

Laryngeal tuberculosis is a rare presentation of tuberculosis. It can mimic laryngeal carcinoma with its clinical and imaging findings. A 51-year old woman underwent ^18^F-fluorodeoxyglucose positron emission tomography/computed tomography (PET/CT) imaging for clinically suspected carcinoma of the larynx. PET/CT revealed lung lesions consistent with tuberculosis in additional to hypermetabolic focus on larynx. The patient was histopathologically diagnosed with lung and laryngeal tuberculosis.

## Abstract (tr)

Laringeal tüberküloz, tüberkülozun nadir bir formudur. Klinik ve radyolojik bulguları larinks karsinomunu taklit edebilir. Klinik olarak larinks karsinomu şüphesi olan 51 yaşında bir kadın hastaya ^18^F-fluorodeoksiglukoz pozitron emisyon tomografisi/bilgisayarlı tomografi (PET/BT) ile görüntüleme yapıldı. PET/BT larinksteki hipermetabolik odağa ilave olarak tüberkülozla ilişkili akciğer lezyonlarını ortaya çıkardı. Hasta histopatolojik olarak akciğer ve larinks tüberkülozu tanısı aldı.

## Introduction

Laryngeal tuberculosis is an infrequent manifestation of extrapulmonary tuberculosis. It occurs in only 1% of all cases (1,2). Usually, it is seen as a complication of pulmonary tuberculosis, nevertheless, solitary laryngeal involvement is possible. Clinical, laryngoscopic and radiological findings of laryngeal tuberculosis have a tendency to mimic laryngeal cancer (3,4). There are no specific findings of extrapulmonary tuberculosis in ^18^F-fluorodeoxyglucose (^18^F-FDG) positron emission tomography/computed tomography (PET/CT), which can also mimic malignancy (5). Herein we present a case of laryngeal tuberculosis who underwent ^18^F-FDG PET/CT imaging with a preliminary diagnosis of laryngeal carcinoma without any clinical pulmonary manifestations.

## Case Report

A 51-year-old woman was referred to our otolaryngology clinic with a history of cough, hoarseness, and sore throat. Her prior medical history was unremarkable. She had been smoking for the past 20 years. The laryngoscopy revealed diffuse swelling and a lesion involving both arytenoids and the marginal portion of the epiglottis, which suggested carcinoma of the larynx. Histopathologic examination of the lesion demonstrated necrosis and was interpreted as suspicious for malignancy, thus recommending a second biopsy. 

**Figure 1:**
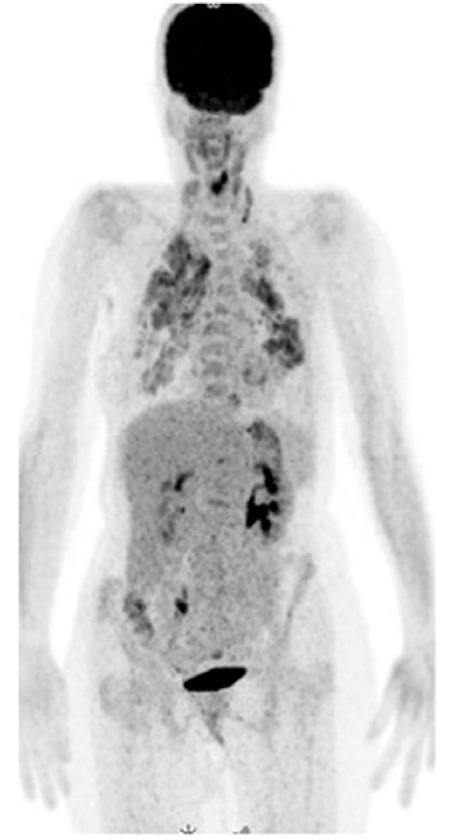
Whole body ^18^F-FDG PET/CT imaging revealed high FDG accumulation in the larynx, lung parenchyma and milimetric cervical lymph nodes

**Figure 2:**
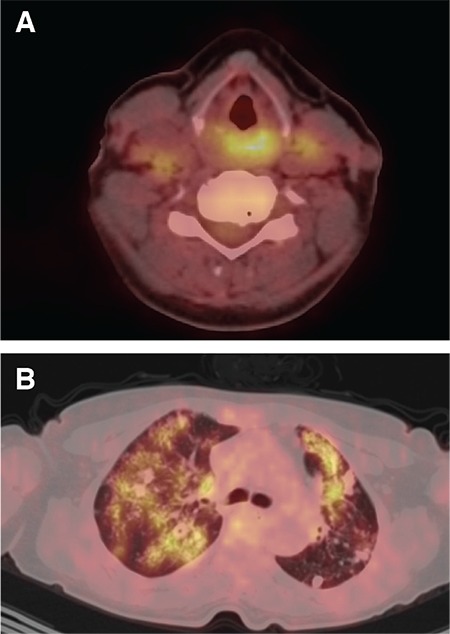
PET/CT fusion images showed hypermetabolic foci in the left aryepiglottic fold and interarytenoid area maximum standard uptake values (SUV_max_): 8.9 (A). On transaxial thorax fusion images, there were multiple hypometabolic nodules (SUV_max_: 1.5) and hypermetabolic infiltrations (SUV_max_: 6) on both lungs, indicating tuberculosis (B)

**Figure 3:**
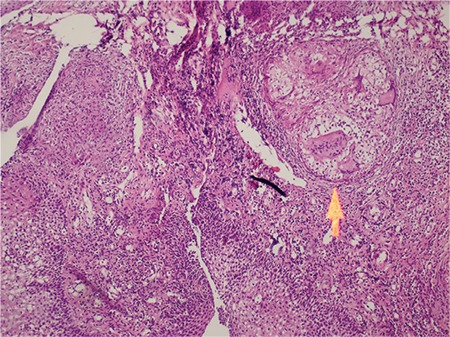
Photomicrograph showing giant cell granuloma (hematoxylin&eosin x200)

Contrast-enhanced computed tomography (CT) scan of the neck demonstrated edema and asymmetry of the epiglottic vallecula. Thorax CT showed multiple nodules that resembled pulmonary metastases on both lungs. The patient underwent ^18^F-FDG PET/CT imaging for diagnosis and staging. PET/CT imaging showed hypermetabolic focus on left aryepiglottic fold and interarytenoid area maximum standard uptake values (SUV_max_): 8.9 without any anatomical correlation. In addition, there were multiple hypometabolic nodules (SUV_max_: 1.5) and hypermetabolic infiltrations (SUV_max_: 6) on both lungs along with mildly hypermetabolic cervical lymph nodes (Figure 1, 2). The second laryngeal biopsy revealed necrotizing granulomatous inflammation suggesting tuberculosis (Figure 3). PCR assay was positive for mycobacterium tuberculosis. The patient was diagnosed as lung and laryngeal tuberculosis, and was started on antituberculosis medication.

## Literature Review and Discussion

Although a rare clinical condition, laryngeal tuberculosis is the most common granulomatous disease of the larynx. Primary laryngeal disease is rare and it usually occurs due to hematogenous dissemination or direct extension of a pulmonary tuberculosis infection (6). The chief complaints in our patient were cough, hoarseness and sore throat. The most common presenting symptom is hoarseness, which is reported to be present in 80-100% of patients. Other symptoms include odynophagia, dysphagia, dyspnea, stridor, cough and hemoptysis (7). These symptoms are also associated with laryngeal carcinoma. On physical examination, laryngeal tuberculosis can manifest as edema, hyperemia, ulcerations, nodule or an exophytic mass. Vocal cords are the most affected site followed by the ventricular strip, epiglottis, subglottic region and posterior commissure (8). CT and MR imaging demonstrate the diffuse nature of the disease and the involvement of the paralaryngeal spaces more accurately than laryngoscopy. Consistent with other studies, Moon et al. (9) detected focal thickening or a mass in the vocal cords, epiglottis and paralaryngeal tissue on CT imaging. ^18^F-FDG PET/CT is a non-invasive imaging method that is being widely used for the differentiation of benign and malignant lesions. However, ^18^F-FDG may also accumulate in inflammatory cells. ^18^F-FDG uptake has previously been reported in tuberculomas and other tuberculosis related lesions (10). In a study, 88 cases with extrapulmonary tuberculosis was reported to show high ^18^F-FDG uptake on PET imaging with a SUV_max_ ranging from 1.3 to 23.2 (11). In our case, PET/CT imaging showed high ^18^F-FDG uptake in the extrapulmonary tuberculosis focus with a SUV_max_ of 8.9. In addition, there were multiple hypometabolic nodules (SUV_max_: 1.5) and hypermetabolic infiltrations (SUV_max_: 6) on both lungs, which were consistent with pulmonary tuberculosis that has not been previously diagnosed. As a whole body scanning method, ^18^F-FDG PET/CT facilitates the detection of extra pulmonary tuberculosis. Although it is a rare condition, extrapulmonary tuberculosis of the head and neck should be kept in mind as part of differential diagnosis, especially in regions where pulmonary tuberculosis is common.
